# P55 The impact of vitamin D deficiency on COVID-19 severity in the UK: a systematic literature review

**DOI:** 10.1093/jacamr/dlaf118.062

**Published:** 2025-07-14

**Authors:** Riya Ann Moncy, Rasha Abdelsalam Elshenawy

**Affiliations:** School of Health, Medicine and Life Sciences, University of Hertfordshire; School of Health, Medicine and Life Sciences, University of Hertfordshire

## Abstract

**Background:**

Vitamin D deficiency affects 20%–40% of the UK population, with higher prevalence among ethnic minorities, older adults, and individuals with obesity.^1^ Deficiency impairs immune responses, increasing susceptibility to respiratory infections such as SARS-CoV-2 [2]. Emerging evidence links low vitamin D levels to greater COVID-19 severity through dysregulated inflammation and impaired ACE2 modulation, although findings remain inconsistent.^3^

**Objectives:**

To evaluate whether vitamin D deficiency independently predicts severe COVID-19 outcomes in UK populations and to examine evidence supporting vitamin D supplementation for prevention or adjunctive therapy.

**Methods:**

Following the PRISMA 2020 guidelines, studies were systematically identified through PubMed, Scopus, and Google Scholar searches covering the period from 2019 to 2025. A comprehensive search strategy was employed, combining terms such as (“vitamin D deficiency” OR “Vit D” OR “25(OH)D”) AND (“COVID-19 severity” OR “SARS-CoV-2”) AND (“UK” OR “United Kingdom”). Studies were independently screened, and quantifiable data on serum vitamin D levels, hospitalization, ICU admission, and mortality were extracted into Excel. Qualitative data regarding mechanisms and UK-specific risk factors were thematically synthesized. Descriptive statistics summarized quantitative findings. Study quality was appraised using the Mixed Methods Appraisal Tool. Ethical approval was not required.

**Results:**

Of the 2030 articles screened, 13 met the inclusion criteria. Two observational studies indicated that vitamin D deficiency (serum levels <50 nmol/L) was associated with a 1.5- to 2.6-fold increased likelihood of severe COVID-19 outcomes (OR: 1.43–2.60). However, analysis of UK Biobank data found no significant adjusted association between vitamin D deficiency and COVID-19 severity (HR: 1.09, 95% CI: 0.91–1.29). Similarly, intensive care studies observed no significant difference in mortality rates between vitamin D-deficient and sufficient patients (*P*=0.64), highlighting inconsistencies in the predictive value of vitamin D deficiency for severe outcomes in UK populations.

**Conclusions:**

This review reveals conflicting evidence regarding vitamin D deficiency and COVID-19 severity in UK populations. While some studies found moderate associations, more robust trials showed no significant benefit after adjusting for confounders. The current evidence does not support disease-specific interventions, though ensuring general vitamin D sufficiency remains essential. Further research should focus on prospective studies with standardized assessments and explore factors such as demographics, comorbidities, and genetic variations to better understand vitamin D's role in COVID-19 outcomes.
